# Modeling Melanoma Heterogeneity In Vitro: Redox, Resistance and Pigmentation Profiles

**DOI:** 10.3390/antiox13050555

**Published:** 2024-04-30

**Authors:** Larissa Anastacio da Costa Carvalho, Isabella Harumi Yonehara Noma, Adriana Hiromi Uehara, Ádamo Davi Diógenes Siena, Luciana Harumi Osaki, Mateus Prates Mori, Nadja Cristhina de Souza Pinto, Vanessa Morais Freitas, Wilson Araújo Silva Junior, Keiran S. M. Smalley, Silvya Stuchi Maria-Engler

**Affiliations:** 1 Department of Tumor Biology, Moffitt Cancer Center, Tampa, FL 33612, USA; larissa.carvalho@moffitt.org (L.A.d.C.C.); keiran.smalley@moffitt.org (K.S.M.S.); 2 Department of Clinical and Toxicological Analysis, School of Pharmaceutical Sciences, University of São Paulo, São Paulo 05508-000, SP, Brazil; isabellanoma@usp.br (I.H.Y.N.); adriana.uehara@usp.br (A.H.U.); 3 Department of Genetics, Ribeirão Preto Medical School, University of São Paulo, Ribeirão Preto 14049-900, SP, Brazil; adamo.siena@yale.com.edu (Á.D.D.S.); wilsonjr@usp.br (W.A.S.J.); 4 Department of Cell Biology and Developmental Biology, Institute of Biomedical Sciences, University of São Paulo, São Paulo 05508-000, SP, Brazil; luciana.osaki@alumni.usp.br (L.H.O.); vfreitas@usp.br (V.M.F.); 5 Department of Biochemistry, Institute of Chemistry, University of São Paulo, São Paulo 05508-000, SP, Brazil; mateus.mori@nih.gov (M.P.M.); nadja@iq.usp.br (N.C.d.S.P.)

**Keywords:** melanoma, heterogeneity, pigmentation, peroxiredoxin 1, peroxiredoxin 2, BRAFi resistance

## Abstract

Microenvironment and transcriptional plasticity generate subpopulations within the tumor, and the use of BRAF inhibitors (BRAFis) contributes to the rise and selection of resistant clones. We stochastically isolated subpopulations (C1, C2, and C3) from naïve melanoma and found that the clones demonstrated distinct morphology, phenotypic, and functional profiles: C1 was less proliferative, more migratory and invasive, less sensitive to BRAFis, less dependent on OXPHOS, more sensitive to oxidative stress, and less pigmented; C2 was more proliferative, less migratory and invasive, more sensitive to BRAFis, less sensitive to oxidative stress, and more pigmented; and C3 was less proliferative, more migratory and invasive, less sensitive to BRAFis, more dependent on OXPHOS, more sensitive to oxidative stress, and more pigmented. Hydrogen peroxide plays a central role in oxidative stress and cell signaling, and PRDXs are one of its main consumers. The intrinsically resistant C1 and C3 clones had lower MITF, PGC-1α, and PRDX1 expression, while C1 had higher AXL and decreased pigmentation markers, linking PRDX1 to clonal heterogeneity and resistance. PRDX2 is depleted in acquired BRAFi-resistant cells and acts as a redox sensor. Our results illustrate that decreased pigmentation markers are related to therapy resistance and decreased antioxidant defense.

## 1. Introduction

Melanoma is the most aggressive type of skin cancer [1,2]. Although BRAF inhibitors (BRAFi) have revolutionized therapy, residual disease persists and relapse is very common [3,4,5,6]. Melanoma is a heterogeneous tumor and BRAFi treatment contributes to the selection of intrinsic and acquired resistant subpopulations [7,8,9]. The key biomarker for this phenotype switching is the melanocyte lineage-specific transcription factor MITF (microphthalmia-associated transcription factor) [10,11]. It has been demonstrated that the MITF^high^/AXL^low^ phenotype is linked to BRAFi sensitivity while MITF^low^/AXL^high^ predicts resistance [12]. MITF is also linked to oxidative metabolism by controlling the transcriptional cofactor PGC-1α (peroxisome proliferator-activated receptor γ coactivator 1), which activates mitochondrial biogenesis and oxidative phosphorylation (OXPHOS) [13,14]. Increased expression of PGC-1α can restore OXPHOS in melanoma and, consequently, lead to higher ROS production by the mitochondria [14,15]. Acquired mutations in BRAF suppresses OXPHOS, MITF, and PGC-1α expression, while BRAFi increases OXPHOS, MITF, PGC-1α, and ROS production. Increased ROS levels makes the tumor more sensitive to oxidative stress [16,17], rendering cells highly dependent on antioxidant defense. BRAFi-resistant cells frequently have adaptive antioxidant mechanisms to tolerate ROS [18]. PGC-1 coactivators are regulators of melanin production via regulation of MITF and melanin production itself is an oxidative process that generates ROS [19,20]. It is known that PGC-1s regulates broad protective programs against ROS [21] and MITF has also been implicated in controlling ROS [22]. Coregulation of melanogenesis and an anti-ROS program by PGC-1s may thus be critical for protecting activated melanocytes from ROS damage [13]. However, the role of melanin on metastasis and resistance remains poorly explored at the heterogeneity level [23].

Transient production of ROS is necessary for cell signaling, but persistent production could be cytotoxic. In this sense, cancer cells require increased levels of ROS for continuous proliferation, but overexpress cellular peroxidases such as peroxiredoxins (PRDXs) to maintain homeostasis [24]. PRDXs are antioxidant enzymes that react with ROS and RNS, and they play a central role in redox signaling acting as redox sensors [25,26,27]; also, the oxidation state of PRDXs, provides information about redox homeostasis [28,29]. Moreover, PRDXs are differentially expressed in patients during melanoma progression [30] and decreased expression of PRDX1 and PRDX2 is a biomarker in melanoma compared to nevus [31]. Additionally, the role of PRDX2 in preventing invasion in BRAF-mutant melanoma has been described in a heterogenous population [32], but its roles in heterogeneity, resistance, and pigmentation are not known [31]. In this study, we investigated clonal heterogeneity and demonstrated a link among pigmentation, resistance, and oxidative stress that could help better understand therapy failure.

## 2. Materials and Methods

### 2.1. Human Melanoma Cell Culture

The human melanoma cell line (SK-MEL-28) was kindly donated by Dr. Maria S. Soengas (Melanoma Group, CNIO, Spain). The SK-MEL-28 BRAFi-resistant cells (SK-MEL-28R) were generated using vemurafenib (PLX4032) as previously described [33]. STR profiling was performed in naïve cells. Cells were cultured in DMEM supplemented with 10% FBS and antibiotics at 37 °C, 5% CO_2_ and humidified atmosphere. All cells were tested for mycoplasma.

### 2.2. Clonal Subpopulations Isolation

Stochastic isolation was performed by seeding naïve SK-MEL-28 one cell per well. Cells were cultivated until confluence was reached and three distinct clones were isolated, called C1, C2, and C3.

### 2.3. Cell Proliferation

Cells (1 × 10^4^ cells/well) were harvested at times 24, 72, 120, and 168 h, and the viable cells were counted using trypan blue in a Neubauer chamber. Doubling times were obtained by non-linear regression analysis with GraphPad Prism 5 (La Jolla, CA, USA).

### 2.4. Wound Healing Assay

The cell (5 × 10^4^ cells/well) monolayer was scratched using a pipette tip after reaching more than 90% confluence and maintained in DMEM containing 1% FBS. Images were taken at 0, 12, and 24 h until the control wound was fully closed. Images were processed and quantified in ImageJ 8 (Bethesda, MD, USA). Results represent the measurements of each wounded area in three independent experiments.

### 2.5. Evaluation of Intrinsic BRAFi Resistance

Cells (2 × 10^4^ cells/well) were treated with 0, 1, or 6 μM of BRAFi. At times 24, 48, and 72 h, viable cells were counted using trypan blue in a Neubauer chamber.

### 2.6. Colony Formation Assay

Cells (1000 cells/well) were treated with 0, 1, and 6 μM BRAFi every 2–3 days for 4 weeks. They were then washed with PBS, fixed, and stained with violet crystal solution for 10 min at RT.

### 2.7. Protein Levels by Western Blot

Cells were lysed with RIPA buffer (Tris 10 mM pH 7.5, NaCl 150 mM, NP 40 1%, SDS 0.1%, Sodium Deoxycolate 1%, EDTA 5 mM, EGTA 5 mM, NaF 25 mM and Na_2_VO_4_ 1 mM) and a mixture of protease and phosphatase inhibitors. For non-reducing Western blot, N-ethylmaleimide 30 mM was added. Proteins were quantified by Bradford, separated by SDS-PAGE under reducing or non-reducing buffer, transferred to a PVDF membrane, and incubated for 1 h at RT with blocking solution (BSA 5%). Then, the membrane was incubated overnight at 4 °C with the primary antibody (1:1000). Next day, the membrane was incubated for 1 h with the secondary antibody (1:2500) and protein bands detected by an ECL system (Luminata^TM^ Forte Western HRP Substrate) (Millipore Corporation, FL, USA) in an Amersham Imager 680 device. Relative densitometry was semi-quantified by ImageJ.

### 2.8. mRNA Levels by RT-qPCR

RNA was extracted with the RNeasy Plus Mini Kit (Qiagen, Hilden, Germany). Purity was evaluated by absorbance at 260/280 and 260/230 nm. The cDNA was obtained by ThermoScript^TM^ RT-PCR System kit from Invitrogen and amplified with the Taqman Mix^®^ Master and Real Time PCR Assays (Thermo Fischer, Waltham, MA, USA). Transcriptions were normalized with β-actin levels in the samples using the Real-Time PCR System from Applied Biosystems^TM^ StepOne^TM^. Relative expression was calculated using the ∆∆Ct method [34].

### 2.9. Oxygen Consumption Rate (OCR)—Seahorse XF24 Flux Analyzer

OCR was measured in intact cells using the XF24 Extracellular Flow Analyzer (Seahorse Bioscience, North Billerica, MA, USA). Cells (3 × 10^4^ cells/well) were plated in XF24 V7 cell culture microplates. After 12–16 h, XF test medium supplemented with D-glucose 10 mM, sodium pyruvate 1 mM, and GlutaMAX^TM^ (Gibco^TM^) (Thermo Fischer, Waltham MA, USA)2 mM was added and incubated in a free-of-CO_2_ atmosphere at 37 °C for 1 h. Respiratory parameters were analyzed based on the protocol for the Seahorse XF cell stress test kit with minor modifications. Three background measurements were performed before the addition of mitochondrial inhibitors. Sequential additions of 1.5 μM oligomycin, 30 μM dinitrophenol (DNP), and 1 μM rotenone plus antimycin A were made. The results are shown relative to the background.

### 2.10. Redox State Assessment by Dihydrorhodamine 123 (DHR) Probe

Cells (5 × 10^4^ cells/well) were incubated in PBS containing 1 g/L of glucose and the DHR fluorescent probe (ThermoFischer, D23806, 10 μM) was added. After 3 h, fluorescence was read (Synergy, Biotek, VT, USA) with excitation at 485 nm and emission at 528 nm.

### 2.11. Evaluation of Hydrogen Peroxide Cytotoxicity

Cells (2 × 10^4^ cells/well) were treated with hydrogen peroxide (0, 50, 100, 200, 300, and 400 μM) for 3 h. Hydrogen peroxide was quantified at 240 nm (ε_240nm_ = 43.6 M^−1^cm^−1^). MTT solution (3-(4,5-dimethylthiazol-2-yl)-2,5-diphenyltetrazolium bromide, 1 mg/mL) was added and cells incubated for 3 h. Reduced formazan was read at 570 nm (BioTek Instruments, Winooski, VT, USA). IC50 values were obtained by non-linear regression analysis with GraphPad Prism 5.

### 2.12. Evaluation of Gliotoxin (GT) Cytotoxicity

Cells (2 × 10^4^ cells/well) were treated with 0, 100, 200, 350, and 500 nM GT (Sigma-Aldrich G9893) (Sigma-Aldrich, Sao Paulo, Brail). Viable cells were counted using trypan blue in a Neubauer chamber. IC50 values were obtained by non-linear regression analysis with GraphPad Prism 5.

### 2.13. Reconstructed Human Skin

Normal human skin cells were obtained from donated foreskin samples from the University of São Paulo Hospital (HU/USP) (CAAE:76737917.5.1001.0067). Cells were isolated as described before [33]. Fibroblasts were cultured in DMEM with antibiotics and 10% FBS; melanocytes were cultured in medium 254; and keratinocytes were cultured in KBM medium. The reconstructed human skin containing melanoma cells was prepared as described previously [33]. Briefly, fibroblasts (1.5 × 10^4^ cells/skin) were mixed in type I collagen and incubated at 37 °C for polymerization of the dermal matrix. Then, keratinocytes (2.5 × 10^4^ cells/skin), melanocytes (0.8 × 10^4^ cells/skin), and melanoma cells (50 × 10^4^ cells/skin) were added. In the next day, skins were transferred to an air–liquid interface for epithelial differentiation for 10 days.

### 2.14. Hematoxylin and Eosin

Reconstructed skins were paraffin included, sectioned (5 μm), and stained with H&E for morphological analysis. All images were obtained by optical microscopy and analyzed by the NIS Elements software 5.02 (Melville, NY, USA).

### 2.15. Immunofluorescence

Slides were deparaffinized, submitted to antigen retrieval with 10 mM sodium citrate, and washed with PBS. A quantity of 10% goat serum was used to prevent unspecific binding of antibodies, followed by incubation with primary antibodies. Sections were incubated with secondary antibodies for 1 h. Slides were mounted in SlowFade antifade mounting with DAPI (Thermo Scientific) for nuclear counterstaining. All images were acquired with a fluorescence microscope (Axio observer, Zeiss, Oberkochen, Germany) using ZEN software 3.4.

### 2.16. In Silico Analysis

Public data were used from 472 samples of primary and metastatic melanoma of the TCGA (*The Cancer Genome Atlas*), which were downloaded from the repository of the University of Carolina Santa Cruz—UCSC (https://xena.ucsc.edu/) (accessed on 1 February 2020). Samples were separated in reference to their expression values normalized by the first and last quartile as “low” and “high”, respectively. The expression values of the gene of interest were verified for these groups and then *boxplots* were generated with the results. The statistical analysis corresponds to the *two-tail* T test among the samples presented.

### 2.17. cBioPortal Database Analysis

To analyze the integrative relationships of the genes and their clinical characteristics in skin cutaneous melanoma, the cBioPortal for Cancer Genomics was used. The cBioPortal for Cancer genomics is an open access resource (http://www.cbioportal.org/) (accessed on 5 February 2020) [35,36]. The terms “MITF”, “PPARGC1A”, “SOX10”, “PRDX1”, “PRDX2”, and “AXL” were searched for in the cBioPortal database for skin cutaneous melanoma (TCGA, Firehose Legacy), n = 479 samples. The selected genomic profiles were mRNA expression and protein expression with a z-score threshold of 1.5.

### 2.18. scRNAseq Analysis

The analysis of single-cell datasets was performed using Interactive Single-Cell Visual Analytics (ISCVA), a computational tool consisting of two major components. The first is a collection of Bash and R scripts that uses many of the widely used algorithms in single cells, such as Seurat for general processing [37], SingleR for cell-type recognition [38], and single-cell signature explorer for gene set signature scoring [39], which processed the scRNA-Seq data offline. The second is a web-based component including react.js from Facebook, tensorflow.js from Google, and Plotly.js. These components allow real-time interactive exploration and ad hoc analysis. As a part of the analytical modules, the heterogeneity analyses implemented in SinCHet [40] were also performed. A node.js backend was created to serve the on-demand queries of the web application, allowing for real-time interactive investigation of genes expressed in selected samples or subsets of cells. Data analyzed are from previous published work (GSE174401 and GSE77940) [41,42].

### 2.19. Statistical Analysis

Results are expressed as mean ± standard error of three independent experiments. One-way analysis of variance (ANOVA) was used followed by the Newman–Keuls post-test to test for multiple comparisons with a given significance level of *p* < 0.05. Alternatively, the *t*-test was used when indicated. Significant differences between the control and treated groups are indicated by * *p* > 0.05, ** *p* > 0.01, and *** *p* > 0.001. All experiments were performed in independent triplicates. All data analyzed and images generated were obtained using the R platform and Prism GraphPad 5.

## 3. Results

### 3.1. Clones Exhibited Distinct Morphology, Proliferation Rate, Intrinsic Resistance, Migration, and Invasion Capacity

**Figure 1:**
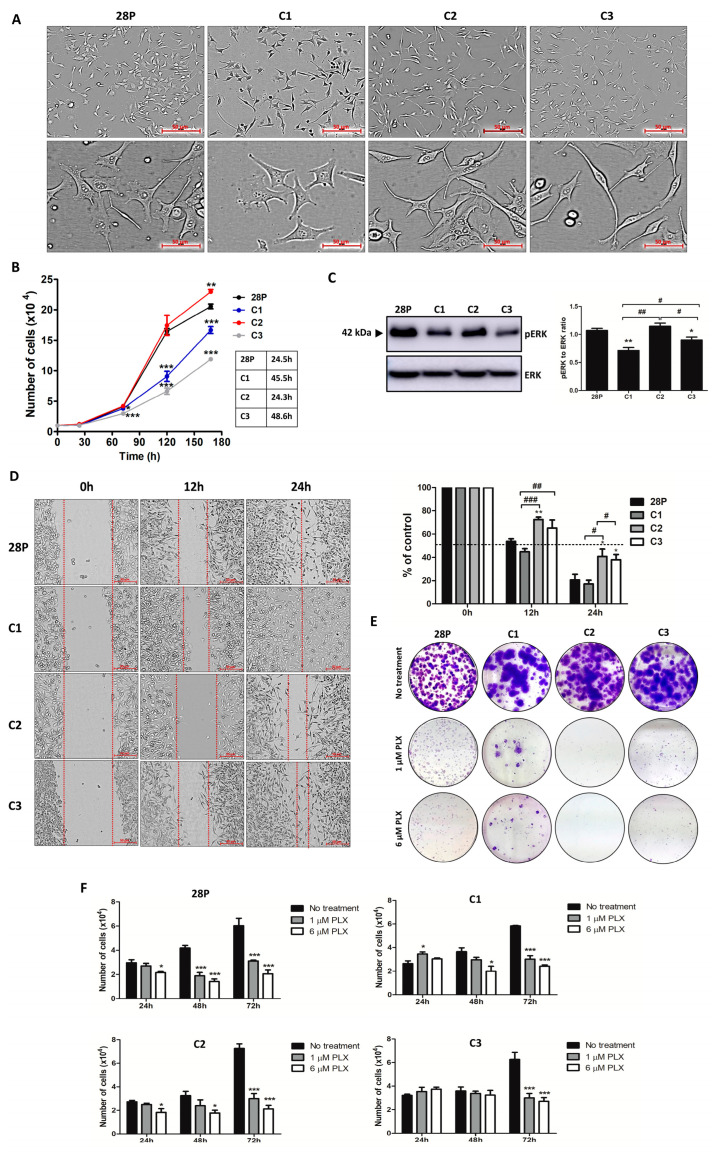
The isolated clones exhibited distinct morphology, proliferation rate, migration capacity, and intrinsic resistance. (**A**) Cell morphology. Scale 100 μm, magnification 10× (above) and 40× (below). (**B**) Proliferation rate. The cells (1 × 10^4^ cells/well) were plated and the viable ones were counted with trypan blue exclusion after 24, 72, 120, and 168 h. (**C**) Evaluation of proliferation by expression of pERK and ERK. (**D**) Wound healing assay. The cells (2 × 10^5^ cells/well) were plated, and after the scratch, photos were taken at 0, 12, and 24h. Scale 50 μm, magnification 10×. (**E**) Colony formation assay. The cells (1000 cells/well) were treated with PLX (0, 1, and 6 µM) for 3 weeks and stained with crystal violet. (**F**) Intrinsic resistance to BRAFi. The cells (2 × 10^4^ cells/well) were plated and treated with PLX (0, 1 and 6 µM), and the viable ones were counted with trypan blue exclusion after 24, 48, and 72 h. All the images are representative of three independent experiments. The values are indicated by mean ± standard error of three independent experiments. The statistical analysis was performed by one-way analysis of variance (ANOVA) followed by a Newmann–Keuls test; *** *p* < 0.001, ** *p* < 0.01 and * *p* < 0.05 when compared to control and # when compared among the groups. 28P: SK-MEL-28 parental cell line. PLX: vemurafenib (BRAFi) (### *p* < 0.001, ## *p* < 0.01, and # *p* < 0.05).

Clone C1 is more spherical than C2 and C3, while clone C3 showed a more elongated shape. A mixture of these morphologies can be seen in the parental cell line (Figure 1A). Clone C2 (24.3 h) and SK-MEL-28P (24.5 h) are more proliferative than C1 (45.5 h) and C3 (48.6 h, Figure 1B). Confirming our results, the phosphorylation of ERK is decreased in C1 and C3 (Figure 1C). Clone C2 seems to be the less migratory than the others (Figure 1D). After a long-term treatment (3 weeks) with BRAFi (acquired resistance), C1 and C3 were capable of surviving and forming viable colonies, unlike C2 (Figure 1E). We tested the intrinsic resistance by short-term treatment with 24, 48, and 72 h of BRAFi exposure and demonstrated that the SK-MEL-28P and C2 were sensitive at 24 h, while C1 and C3 needed 48h to respond (Figure 1F).

**Figure 2:**
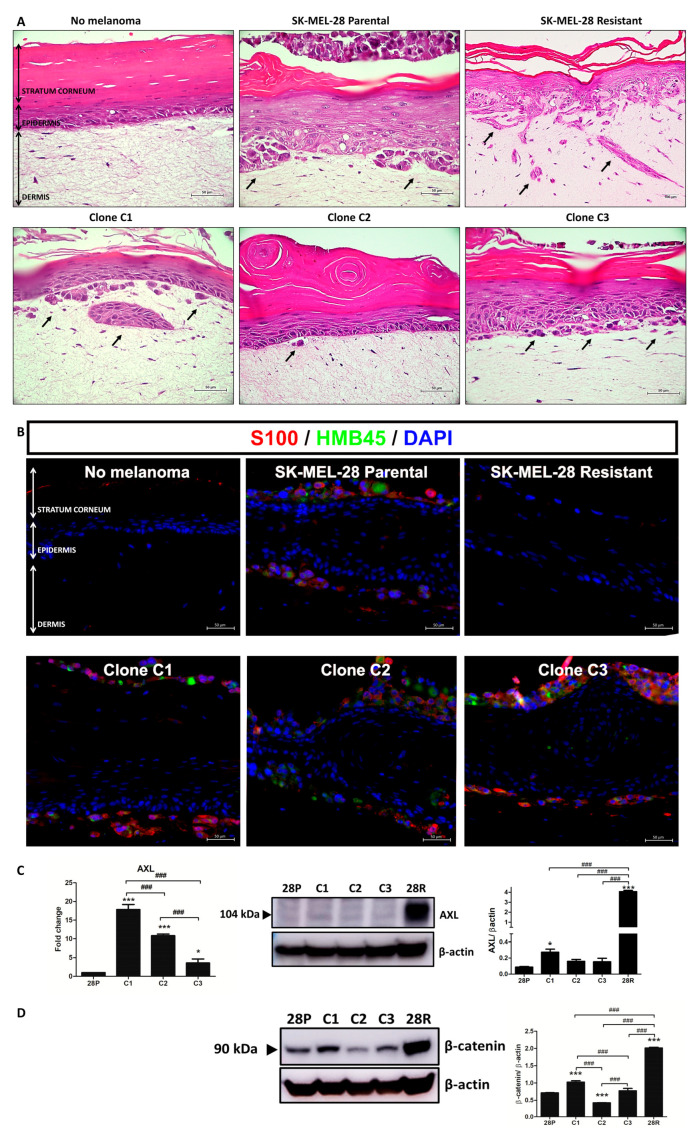
Clones exhibited distinct invasion profiles. (**A**) Human reconstructed skin containing no melanoma, SK-MEL-28 parental, clone C1, clone C2, clone C3, or SK-MEL-28 resistant stained for H&E. Scale 50 μm, magnification 40×. (**B**) Immunofluorescence for human reconstructed skin containing no melanoma, SK-MEL-28 parental, clone C1, clone C2, clone C3, or SK-MEL-28 resistant stained for S100 and HMB45. Scale 50 μm, magnification 40×. (**C**) mRNA and protein levels of AXL in SK-MEL-28 parental, clones, and SK-MEL-28R. (**D**) Protein levels of beta-catenin in SK-MEL-28 parental, clones and SK-MEL-28R. 28P: SK-MEL-28 parental cell line. 28R: SK-MEL-28-resistant cell line. The statistical analysis was performed by one-way analysis of variance (ANOVA) followed by a Newmann–Keuls test; *** *p* < 0.001 and * *p* < 0.05 when compared to control and # when compared among the groups (### *p* < 0.001).

In a 3D skin model, SK-MEL-28P invaded the dermis and proliferated on the corneal layer (Figure 2A). The SK-MEL-28R also invaded the dermis even in the presence of BRAFi. Among the clones, C1 showed the most invasive profile, followed by C3 and C2. We confirmed the melanoma invasion within the dermis with S100 and HMB45 staining in Figure 2B. Further studies demonstrated that clone C1 and the SK-MEL-28R cells showed higher levels of AXL and beta-catenin (Figure 2C,D).

### 3.2. Clones Exhibited Distinct Oxidative Metabolism

**Figure 3:**
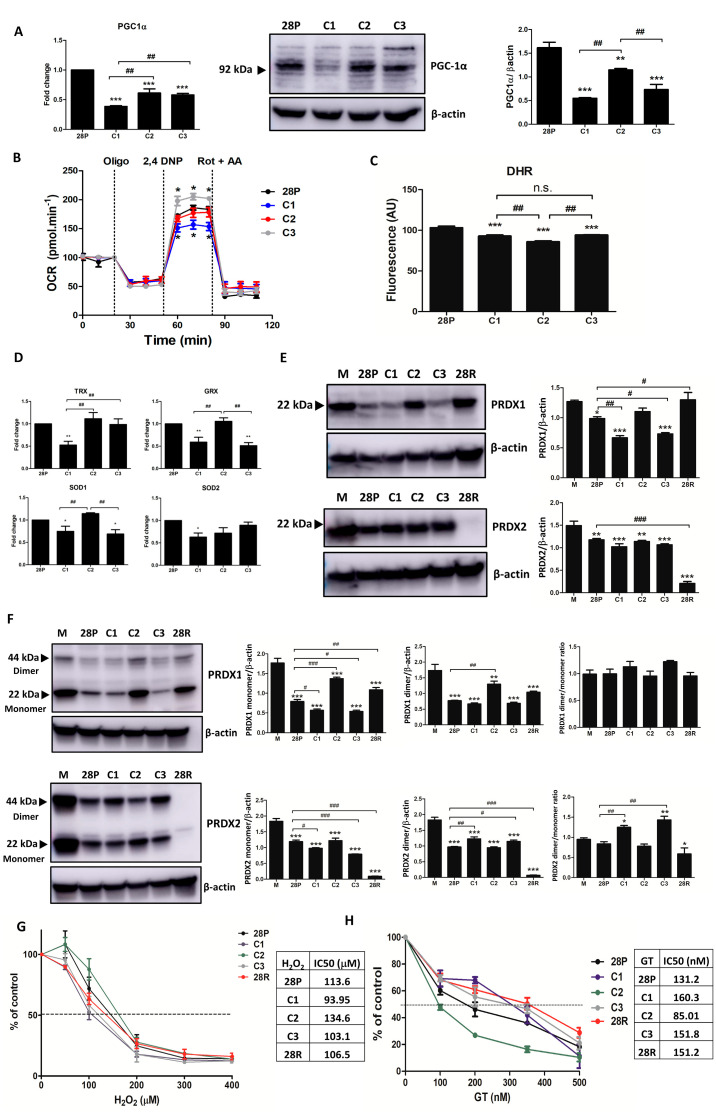
Clones exhibited distinct oxidative metabolism. (**A**) Expression of mRNA and protein of PGC−1α. (**B**) Oxygen consumption rate (OCR). The cells (3 × 10^4^ cells/well) were treated with indicated drugs (oligomycin 1.5 μM, 2,4-dinitrophenol 30 mM, rotenone 1 μM, and antimycin A 1 μM) at the determined times. The values are indicated by mean ± standard error of three independent experiments. Statistical analysis was performed by *t*-test comparing with the parental SK-MEL-28P control, * *p* < 0.05. (**C**) Detection of total reactive oxygen species using DHR probe. The cells (5 × 10^4^ cells/well) were seeded in a black plate, treated with DHR (10 μM) for 3 h and then fluorescence was read (λexc 485 nm and λem 528 nm). The values are indicated by mean ± standard error of three independent experiments. Statistical analysis was performed by one-way analysis of variance (ANOVA) followed by Newmann–Keuls test, *** *p* < 0.001, ** *p* < 0.01, and * *p* < 0.05 compared to parental control (# when compared among groups). (**D**) TRX, GPX, SOD1, and SOD2 mRNA levels in clones. (**E**) Protein levels of PRDX1 and PRDX2 in melanocytes (M), SK-MEL-28 parental, clones, and SKM-MEL-28R. (**F**) Western blot redox for PRDX1 and PRDX2. (**G**) Determination of the sensitivity of the cells to hydrogen peroxide by calculating the IC50. (**H**) Determination of the sensitivity of the cells to gliotoxin by calculating the IC50. The cells (2 × 10^4^ cells/well) were treated with increasing concentrations of hydrogen peroxide (0, 100, 200, 300, and 400 nM). After 24 h and 5 h, viable cells were determined. 28P: SK-MEL-28 parental cell line. 28R: SK-MEL-28-resistant cell line (### *p* < 0.001, ## *p* < 0.01, and # *p* < 0.05).

PGC-1α levels were decreased in all clones, with C1 and C3 showing the lowest levels (Figure 3A). C1 was less dependent on OXPHOS, C3 was more dependent, and C2 resembled the profile of SK-MEL-28P (Figure 3B). Clones C1 and C3 showed higher ROS production compared to C2. The SK-MEL-28P exhibited higher levels of ROS, probably because of the presence of other clones (Figure 3C). The antioxidant system in general was decreased in C1 (Figure 3D). C1 and C3 expressed less PRDX1 when compared to SK-MEL-28P. In addition, SK-MEL-28P and the clones C1 and C3 exhibited decreased expression of PRDX1 when compared to primary human melanocytes, while clone C2 and SK-MEL-28R showed comparable levels to melanocytes (Figure 3E). No differences in PRDX2 levels were seen among the clones (Figure 3E), but PRDX2 was depleted in the SK-MEL-28R while PRDX1 was increased in SK-MEL-28R compared to SK-MEL28P (Figure 3E). Once PRDX dimers were formed after oxidation, non-reducing Western blot was performed to probe oxidative stress. As expected, cells that showed increased levels of PRDX1 also had increased expression of monomers and dimers. We calculated the dimer/monomer ratio to evaluate the redox status [43] (Figure 3F, upper figure) and found no differences among the cells. In contrast, levels of PRDX2 monomers were lower in C1 and C3 while the dimers were higher, providing evidence that these had a more oxidative environment (Figure 3F, lower figure), in agreement with the data obtained with DHR (Figure 3C). Once PRDX1 levels are variable among the clones and PRDX2 levels are maintained, this result suggests that PRDX2, but not PRDX1, could be used as an oxidative stress probe. We then tested the sensitivity of the clones to hydrogen peroxide (Figure 3G) and gliotoxin (GT), an antioxidant and PRDX mimetic (Figure 3H). C1 was the most sensitive to hydrogen peroxide (IC50 = 93.95 μM), followed by C3 (IC50 = 103.1 μM), SK-MEL28R (106.5 μM), SK-MEL-28P (113.6 μM), and C2 (134.6 μM). By contrast, clone C2 was the most sensitive to GT (IC50 = 85.01 nM), followed by SK-MEL-28P (IC50 = 131.2 nM), SK-MEL-28R (IC50 = 151.2 nM), clone C3 (IC50 = 151.8 nM), and clone C1 (IC50 = 160.3 nM). In summary, C1 and C3, which were the most sensitive to oxidative stress, were also the most resistant to GT. So, it seems that C1 and C3 produce more ROS and/or exhibit a weaker antioxidant system compared to clone C2.

### 3.3. Clones Showed Different Profiles of Pigmentation

**Figure 4:**
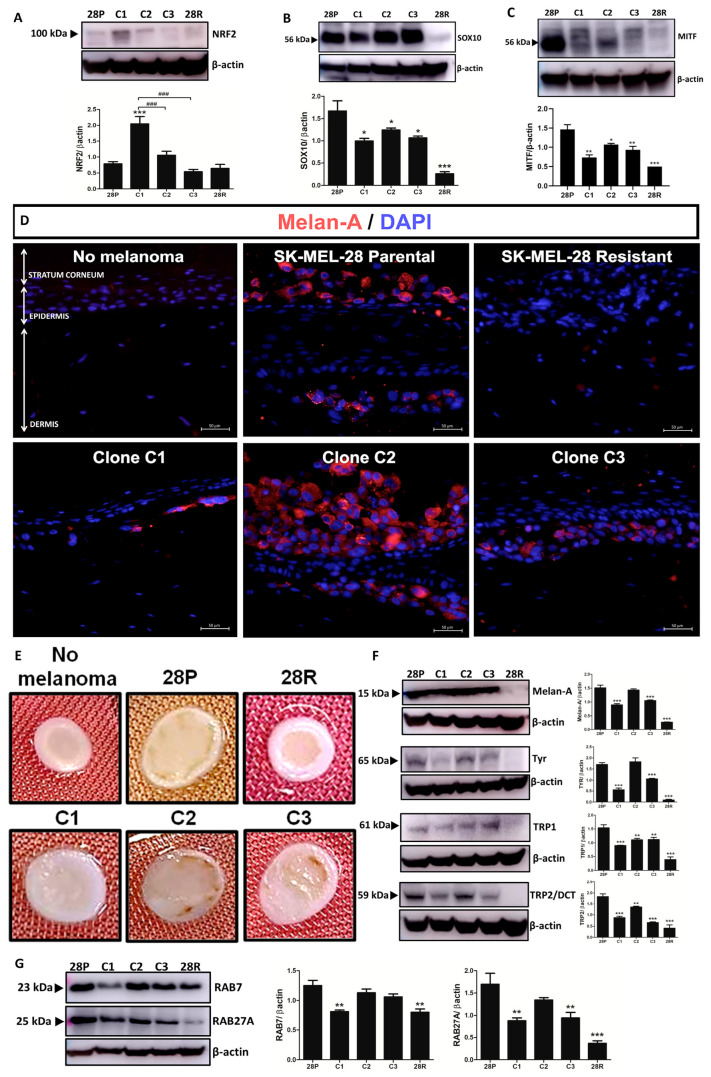
Clones exhibited different patterns of pigmentation. (**A**) Protein levels of NRF2. (**B**) Protein levels of SOX10. (**C**) Protein levels of MITF. (**D**) Immunofluorescence of skin reconstructed containing no melanoma, SK-MEL28 parental, clones, or SK-MEL-28R stained for Melan-A. Scale 50 μm, magnification 40×. (**E**) Images of the reconstructed skins showing the differences in pigmentation. (**F**) Protein levels of Melan-A, TYR, TRP1, and TRP2. (**G**) Protein levels of RAB7 and RAB27. The values are indicated by mean ± standard error of three independent experiments. Statistical analysis was performed by one-way analysis of variance (ANOVA) followed by Newmann–Keuls test, *** *p* < 0.001, ** *p* < 0.01, and * *p* < 0.05 when compared to parental control (# when compared among groups). 28P: SK-MEL-28 parental cell line. 28R: SK-MEL-28-resistant cell line (### *p* < 0.001).

An analysis of melanocyte lineage/pigmentation markers demonstrated that NRF2 expression was significantly increased in clone C1, while SOX10 and MITF were significantly decreased (Figure 4A–C). It was further noted that Melan-A levels were decreased in the skin containing C1 or SK-MEL-28R (Figure 4D). Skins exhibited different patterns of pigmentation with the SK-MEL-28P and the clones C2 and C3 exhibiting the most pigmented, while C1 and SK-MEL28R showed no pigmentation (Figure 4E). We confirmed these results showing that the pigmentation markers Melan-A, TYR, TRP1, and TRP2 were decreased in C1 and SK-MEL-28R (Figure 4F). We also demonstrated that RAB7 and RAB27 were decreased in C1 and SK-MEL-28R (Figure 4G).

### 3.4. In Silico Analysis of PRDX1 and PRDX2 Expression in Melanoma Patients

**Figure 5:**
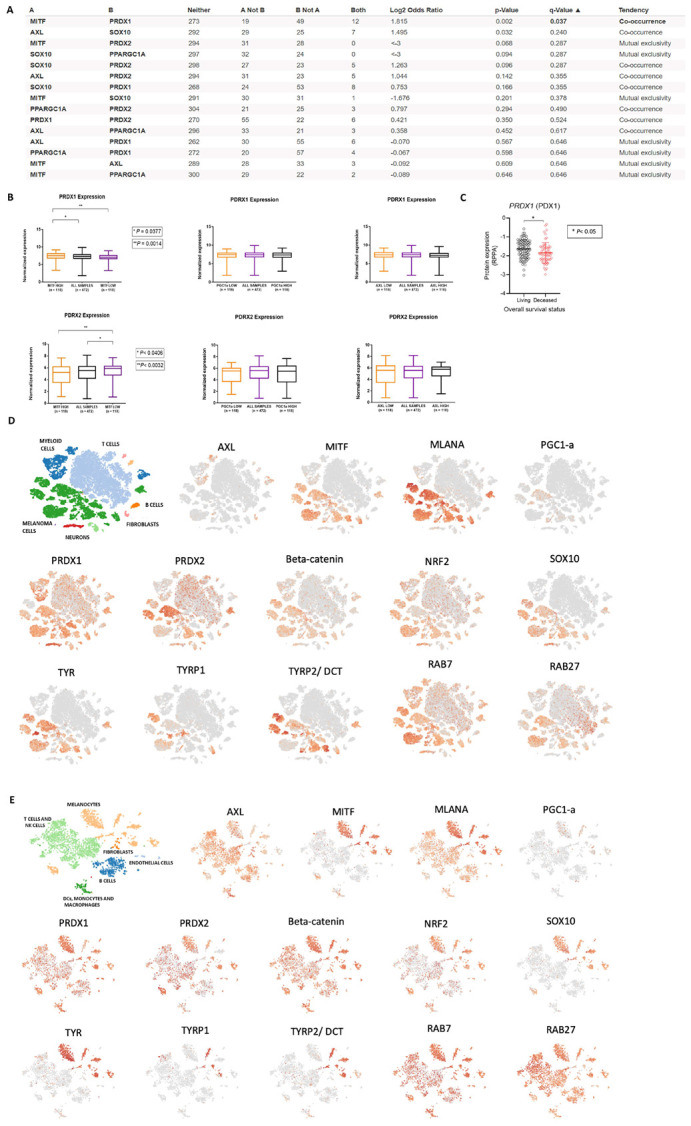
In silico analysis of the expression of PRDX1 and PRDX2 in melanoma patients. (**A**) Correlation between AXL, SOX10, MITF, PGC1−α, PRDX1, and PRDX2 in melanoma patients according to cBioPortal. (**B**) Expression of PRDX1 and PRDX2 in cells expressing high or lower levels of MITF, PGC1-α and AXL. (**C**) Correlation of overall survival and levels of PRDX1. (**D**,**E**) scRNAseq data of proteins involved in proliferation, invasion, metabolism, redox processes, BRAFi, resistance, and pigmentation.

PRDX1 shows significant co-expression with MITF in melanoma patients, with PRDX2 showing a tendency towards mutual exclusivity (Figure 5A). There was also a tendency for co-occurrence between PRDX2 and AXL, whereas PRDX1 and AXL were mutually exclusive (Figure 5A). Patients with low levels of MITF have significantly lower levels of PRDX1 and higher levels of PRDX2, while no differences were found on AXL and PGC1α compared to PRDX1 and PRDX2 (Figure 5B). Additionally, BRAF-mutated patients with decreased expression of PRDX1 demonstrated poor survival rates compared to those with higher expression of PRDX1 (Figure 5C). Using single-cell data from metastatic melanoma patients published by [42], we observed that MITF, SOX10, MLANA, PGC1a, TYR, TYRP1, and TYRP2/DCT are present only or enriched in melanoma cells. Additionally, NRF2 expression was noted in all cell types at a lower expression than that of MITF in melanoma cells. The expression of PRDX1 was higher than that of PRDX2 in melanoma cells and more similar to that of MITF than that of PRDX2. Furthermore, the expressions of TYR, TYRP1, and TYRP2/DCT were similar to those of MITF and MLANA, corroborating our data obtained using the isolated clones, where C1 expresses less MITF, MLANA, TYR, TYRP1, TYPR2/DCT, and PRDX1 (Figure 5D). We also analyzed another dataset published in [41], containing melanoma patients with skin, brain, and leptomeningeal metastasis. As expected, PRDX1 and PRDX2 were distributed across all cell types. However, it was interesting to note that the expressions of PRDX1 and PRDX2 were different among the cells. We found PRDX1 enriched in neurons more than PRDX2, while some populations of melanoma cells express more PRDX2 than PRDX1. Then, PRDX1 expression is similar to MITF in patient melanoma cells, which corroborates our in vitro data using the clones (Figure 5E).

## 4. Discussion

Tumor heterogeneity is one of the causes of treatment resistance and failure [44,45]. It arises from complex genetic, epigenetic, and proteomic/transcriptomic modifications that drive phenotypic selection and alterations in response to microenvironment pressures [46]. In fact, a recent finding showed the potential to predict initial BRAFi sensitivity at the single-cell level [47]. The understanding of driver agents, the functional consequences of heterogeneity, and the transcriptional profiles of the clonal subpopulations may provide new insights that can optimize therapy. MITF regulates phenotype switching since MITF^high^ cells exhibit a proliferative profile, while MITF^low^ cells exhibit a more invasive one [10,11]. These data are in agreement with low MITF expression in C1 and C3. Considering that MITF controls the expression of PGC-1α, clones C1 and C3 also exhibited significant decreased expression of PGC-1α. SOX10 contributes to melanoma development by regulating the SOX10-MITF pathway, but also contributes to melanoma cell survival, proliferation, and metastasis formation [48,49]. AXL is linked to the mesenchymal state in the epithelium–mesenchymal transition, while melanoma cells with low AXL expression resemble the epithelial state [50]. The MITF^high^/AXL^low^ phenotype is linked to BRAFi sensitivity and the MITF^low^/AXL^high^ phenotype is linked to resistance [12], and we confirmed that C1 had the MITF^low^/AXL^high^ profile along with intrinsic resistance to BRAFi. Of relevance, cells that are MITF^low^/AXL^high^ can co-exist with MITF^high^/AXL^low^ cells and MITF^high^ cells, and can cluster with distinct transcriptional states and be drug-sensitive or resistant [47,51].

BRAFi resistance is characterized by high mitochondrial activity that can occur regardless of PGC-1α expression [18]. Our results corroborate previous data and provide new evidence that naïve and intrinsically BRAFi-resistant cells like C1 could also display low mitochondrial activity. The presence of a BRAF mutation is associated with suppression of OXPHOS, MITF, and PGC-1α expression, while BRAFi can increase OXPHOS, MITF, PGC-1α, and ROS production. Additionally, ROS can be increased through a dependent or independent PGC1α mechanism [13,18]. MITF is redox-sensitive, since oxidative injury induced by hydrogen peroxide drives downregulation of MITF and its downstream targets [52]. Additionally, MITF dysfunction may lead to oxidative damage once MITF deficiency in mice leads to higher levels of ROS. Overexpression of MITF upregulates the antioxidant response and mitochondrial biogenesis by regulating PGC-1α [53]. Therefore, MITF is possibly involved in redox signaling by controlling the PGC1α/mitochondria/OXPHOS/antioxidant axis [53]. MITF also controls the cellular response to ROS by regulating APE-1 [22]. Then, MITF^high^ cells were found to be more resistant to hydrogen peroxide-induced cell death [22], which corroborates our data obtained for C2 treated with hydrogen peroxide. It was demonstrated that APE-1/PRDX1 could act as an anti-inflammatory agent avoiding cancer invasion and metastasis [54]. Additionally, the transcription factor NRF2, which is the major mediator of oxidative stress responses and linked to therapy resistance, suppresses MITF activity and reduces the expression of pigmentation markers in melanoma [55].

MITF targets the pigment genes that encode enzymes necessary for melanin synthesis, TYR, TYRP1, and TYRP2, together with Melan-A [56], and PGC-1s are involved in this regulation [13,19]. The role of melanin in melanoma is contradictory, and a clinical study showed that amelanotic melanoma was associated with poorer patient survival than pigmented melanoma [57]; however, pigmentation is also associated with a worse outcome in metastatic melanoma patients and increased resistance to therapy [58]. Some studies demonstrated that melanin is associated with invasion suppression [59,60] and inhibition of metastasis [24,58,61]. Inhibiting melanoma growth and invasion at early stages, when melanogenesis is deregulated at advanced stages, and its intermediates have an immunosuppressive effect [62], can also induce mutations and DNA damage, and can switch energy yielding metabolism [63]. RAB7 expression is induced in early-stage tumors and downregulated in invading melanomas [64,65]. RAB27 was identified as a melanoma driver; its levels increase during melanoma progression and its depletion decreases tumor progression and metastasis [66], and it is also linked with decreased survival [67]. In contrast to RAB27, RAB7 expression is not regulated by MITF and high RAB7 expression was linked to a decrease in invasiveness and an increase in patient survival [67]. C1 had decreased expression of pigmentation markers and RAB7 and RAB27, suggesting that the presence of melanin could inhibit invasion and resistance.

Interestingly, depletion of PGC-1α caused a significant decrease in antioxidant protein expression [14]. We confirmed that C1 and C3 had lower PRDX1, so it is likely that the imbalance in antioxidant defense could lead to increased ROS production, supporting our observation that C1 and C3 exhibit high levels of ROS. ROS can play a dual role in cancer: a tumorigenic role by promoting proliferation and genomic instability [68], or a toxic role by inducing cellular damage and death [69]. Redox signaling can be regulated by redox-sensitive proteins such as thiol-containing proteins, which guarantee the high specificity of signal transduction, in addition to avoiding the accumulation of ROS [70]. PRDXs are thiol-containing enzymes that play an important role in redox signaling associated with tumorigenesis [70]. PRDXs can directly react with transcription factors and are involved in regulation of apoptosis, differentiation, and proliferation [71]. We have previously demonstrated that PRDX1 was increased in melanoma patients from the vertical growth phase compared to common nevus, and was decreased in metastatic melanoma compared to dysplastic nevus, whereas PRDX2 is decreased in the vertical growth phase and in melanoma metastasis when compared to dysplastic nevus [30]. We observed that PRDX1 and PRDX2 levels were decreased in SK-MEL-28P compared to normal melanocytes and PRDX1 is increased in resistance, while PRDX2 is depleted in acquired resistant SK-MEL-28R. Using data from melanoma patients, we observed that low levels of MITF expression are associated with decreased PRDX1 expression. Our results suggested that PRDX1 and PRDX2 may play distinct roles in melanoma heterogeneity.

It was demonstrated that PRDXs can contribute to resistance to therapy [72,73] and act as an oncogene or tumor suppressor, depending on the tumor [74] and the melanoma stage [30]. In melanoma it is accepted that they are downregulated in metastasis [30,31,75]. PRDXs are known to be involved in receptor tyrosine kinase-mediated signal transduction [76] and modulation of phosphokinase signaling cascades [25,77,78], and may play an important role as tumor suppressor [77,79]. On the other hand, increased expression of PRDX1 by ROS can prevent cell death and PRDX1 is involved in melanoma resistance [80,81,82]. Downregulation of PRDX2 in melanoma cells is related to more production of ROS, proliferation, and migration [32]. In fact, it was demonstrated that PRDX2 prevents metastasis by increasing E-cadherin/β-catenin complexes [32], and that PRDX2 is downregulated in melanomas and could be restored by a demethylating agent [83]. GT is a mimetic of PRDX by reducing hydrogen peroxide to water and accelerating NADPH oxidation by the thioredoxin system [84]. Its potent activity has already been demonstrated in different types of cancer [85,86,87,88,89]. In melanoma, its IC50 is 100–200 nM [32,86]. Interestingly, we found an inverse correlation among sensitivity to oxidative stress and antioxidant treatment.

Taken together, our results demonstrated that clones in the same tumor can naturally express less PRDX1, MITF, SOX10, PGC-1α, and pigmentation markers. We linked this genetic profile to a phenotype that is more invasive, less pigmented, and intrinsically resistant. These results provide evidence for the influence of redox-sensitive reactions in melanoma heterogeneity and the role of pigmentation in this context, highlighting the importance of antioxidant response in treatment resistance.

## Data Availability

Data are contained within the article.
